# 14 MeV Neutrons for ^99^Mo/^99m^Tc Production: Experiments, Simulations and Perspectives

**DOI:** 10.3390/molecules23081872

**Published:** 2018-07-27

**Authors:** Marco Capogni, Antonino Pietropaolo, Lina Quintieri, Maurizio Angelone, Alessandra Boschi, Mauro Capone, Nadia Cherubini, Pierino De Felice, Alessandro Dodaro, Adriano Duatti, Aldo Fazio, Stefano Loreti, Petra Martini, Guglielmo Pagano, Micol Pasquali, Mario Pillon, Licia Uccelli, Aldo Pizzuto

**Affiliations:** 1 ENEA-Department of Fusion and Technology for Nuclear Safety and Security, Via E. Fermi 45, Frascati, I-00044 Roma, Italy; marco.capogni@enea.it (M.C.); lina.quintieri@stfc.ac.uk (L.Q.); maurizio.angelone@enea.it (M.A.); mauro.capone@enea.it (M.C.); nadia.cherubini@enea.it (N.C.); pierino.defelice@enea.it (P.D.F.); alessandro.dodaro@enea.it (A.D.); aldo.fazio@enea.it (A.F.); stefano.loreti@enea.it (S.L.); guglielmo.pagano@enea.it (G.P.); mario.pillon@enea.it (M.P.); aldo.pizzuto@enea.it (A.P.); 2 ENEA—Italian National Institute of Ionizing Radiation Metrology (INMRI), Casaccia Research Centre, Via Anguillarese 301, 00123 Roma, Italy; 3 Department of Morphology, Surgery and Experimental Medicine, University of Ferrara, Via Ludovico Ariosto, 35-44121 Ferrara, Italy; alessandra.boschi@unife.it (A.B.); petra.martini@unife.it (P.M.); licia.uccelli@unife.it (L.U.); 4 Department of Chemical and Pharmaceutical Sciences, University of Ferrara, Via Ludovico Ariosto 35, 44121 Ferrara, Italy; adriano.duatti@unife.it; 5 Department of Physics and Earth Sciences, University of Ferrara, Via Ludovico Ariosto 35, 44121 Ferrara, Italy; micol.pasquali@unife.it

**Keywords:** Technetium-99m, Molibdenum-99, neutron generator

## Abstract

Background: the gamma-emitting radionuclide Technetium-99m (^99m^Tc) is still the workhorse of Single Photon Emission Computed Tomography (SPECT) as it is used worldwide for the diagnosis of a variety of phatological conditions. ^99m^Tc is obtained from ^99^Mo/^99m^Tc generators as pertechnetate ion, which is the ubiquitous starting material for the preparation of ^99m^Tc radiopharmaceuticals. ^99^Mo in such generators is currently produced in nuclear fission reactors as a by-product of ^235^U fission. Here we investigated an alternative route for the production of ^99^Mo by irradiating a natural metallic molybdenum powder using a 14-MeV accelerator-driven neutron source. Methods: after irradiation, an efficient isolation and purification of the final ^99m^Tc-pertechnetate was carried out by means of solvent extraction. Monte Carlo simulations allowed reliable predictions of ^99^Mo production rates for a newly designed 14-MeV neutron source (New Sorgentina Fusion Source). Results: in traceable metrological conditions, a level of radionuclidic purity consistent with accepted pharmaceutical quality standards, was achieved. Conclusions: we showed that this source, featuring a nominal neutron emission rate of about 10^15^ s^−1^, may potentially supply an appreciable fraction of the current ^99^Mo global demand. This study highlights that a robust and viable solution, alternative to nuclear fission reactors, can be accomplished to secure the long-term supply of ^99^Mo.

## 1. Introduction

**Figure 1:**
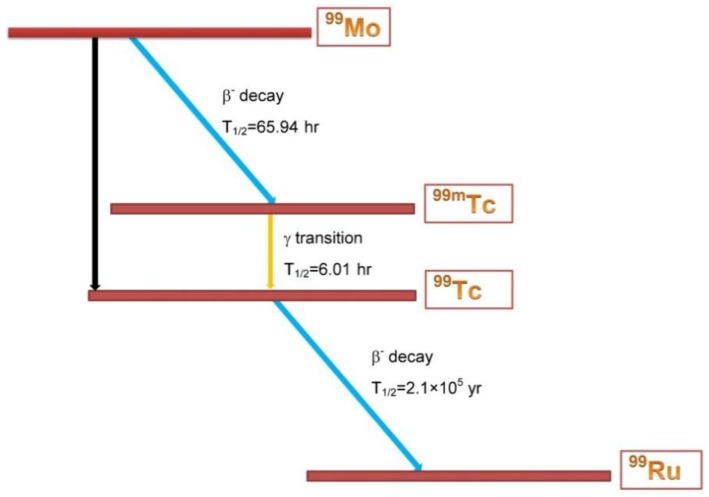
(color online) Decay scheme of ^99^Mo: The black arrow indicates the transition of ^99^Mo directly to the ^99^Tc ground state; blue arrows indicate the transition from ^99^Mo to ^99m^Tc and from ^99^Tc ground state to ^99^Ru. The yellow arrow indicates the transition from ^99m^Tc to ^99^Tc.

Metastable Technetium-99 (^99m^Tc) is a well-suited radionuclide for medical imaging thanks to its short half-life [1] T_1/2_ = 6.0067(10)h [2] and the 140 keV γ-ray emission. Radiopharmaceuticals based on ^99m^Tc are used worldwide in Single Photon Emission Computed Tomography, with more than 30 million procedures per year, accounting for about 85% of all nuclear medicine diagnostics [3]. Nuclear medicine departments in hospitals usually use ^99m^Tc as extracted from ^99^Mo/^99m^Tc generators, where ^99^Mo, whose half-life is T_1/2_ = 2.77479(6)d [4], acts as ^99m^Tc precursor, as shown in Figure 1. This makes possible the delivery of the generators at long distances from the production sites. At present, ^99^Mo is almost exclusively obtained from the fission of ^235^U-containing targets, irradiated in a small number of research nuclear fission reactors in the world [5]. A realistic estimation of the weekly ^99^Mo activity demand at world level, expressed in terms of 6-days Curie (6-day Ci), is about 444 TBq. The 6-days Ci is the ^99^Mo activity available 6 days after the so-called end of target processing (EOP) [3].

A global shortage of ^99^Mo emerged in the late 2000s because of the frequent shut down due to extended maintenance periods of the main reactors for ^99^Mo production, namely the Chalk River National Research Universal (NRU) nuclear fission reactor in Canada and the High Flux Reactor (HFR) in the Netherlands. These are capable of meeting about two-thirds of ^99^Mo world demand [6]. These events highlighted vulnerabilities in the standard production/supply chain of medical radionuclides that relies on nuclear fission reactors. 

The scientific community then became aware of a forthcoming world level problem in ^99^Mo/^99m^Tc production methods and supply, as it is well described and outlined in different papers [6,7]. International organizations such as the International Atomic Energy Agency (IAEA) and the Organization for Economic Co-operation and Development (OECD) have been working to identify specific action guidelines for the production of ^99^Mo.

Three general methods of ^99m^Tc production (direct or by means of ^99^Mo precursor) have been identified as short-, mid- and long-term alternative solutions to the reactor-based ^235^U fission technique [5]:

- Thermal or fast (14 MeV) neutron beams: ^98^Mo(n,γ)^99^Mo and ^100^Mo(n,2n)^99^Mo, respectively.
- Gamma-ray beam: ^100^Mo(γ,n)^99^Mo.
- Accelerated charged-particle beams: ^96^Zr(α,n)^99^Mo or ^100^Mo(p,2n)^99m^Tc.

Here, we thoroughly investigate the ^100^Mo(n,2n)^99^Mo reaction induced by 14 MeV neutrons, having been identified [8,9] as a possible alternative method to ^235^U fission. Indeed, the ^100^Mo(n,2n)^99^Mo cross section is marked and exhibits a maximum of about 1.5 barn around 14 MeV.

As indicated in official documents [10], the method based on ^100^Mo(n,2n)^99^Mo can be a valuable and promising route to ^99^Mo production as it also minimizes issues related to nuclear waste, that represent one of the main concerns for nuclear fission reactors. On the other hand, the effective ^99^Mo quantity achievable with a 14 MeV neutron source must be clearly and quantitatively assessed, to demonstrate to what extent this alternative way may be a viable solution for a global level production.

In this respect, we perform experiments irradiating a metallic natural molybdenum powder with 14 MeV neutrons generated in deuteron-tritium (D-T) fusion reactions at the Frascati Neutron Generator (FNG), operating at the ENEA Frascati Research Centre [11,12]. In preparation of the experiment, we perform Monte Carlo (MC) simulations of the irradiation tests using the Fluka code [13]. The simulated experiment is benchmarked by experimental data, allowing the provision of quantitative predictions of the ^99^Mo production by means of the New Sorgentina Fusion Source (NSFS) [14,15,16], a very intense 14 MeV D-T neutron source under investigation at ENEA, and currently in an advanced conceptual design stage. 

## 2. Methods

### 2.1. ^100^Mo(n,2n)^99^Mo Reaction with 14 MeV Neutrons

The nuclear reaction involved in the production of ^99^Mo with 14 MeV neutrons is a process that can be described as n + M42100o→ M4299o + 2n.

**Figure 2:**
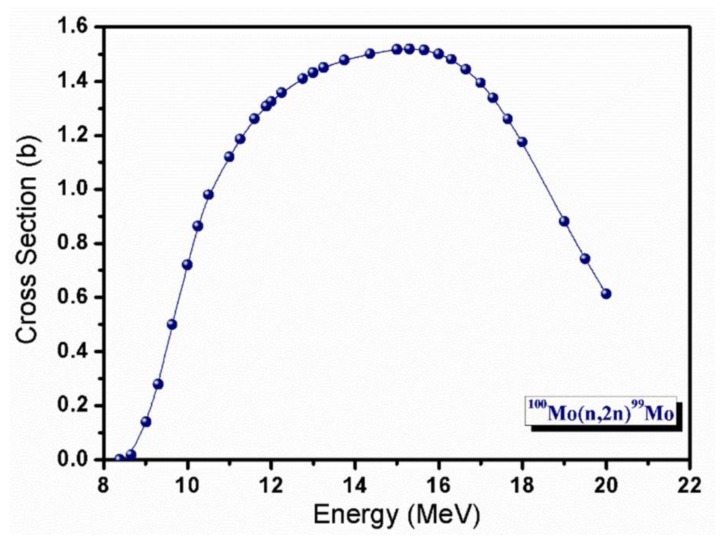
Cross section of the inelastic nuclear reaction ^100^Mo(n,2n)^99^Mo.

The cross section of this process has a broad maximum around 14 MeV and a threshold at about 8 MeV (Figure 2) [17].

#### 2.1.1. The Frascati Neutron Generator

**Figure 3:**
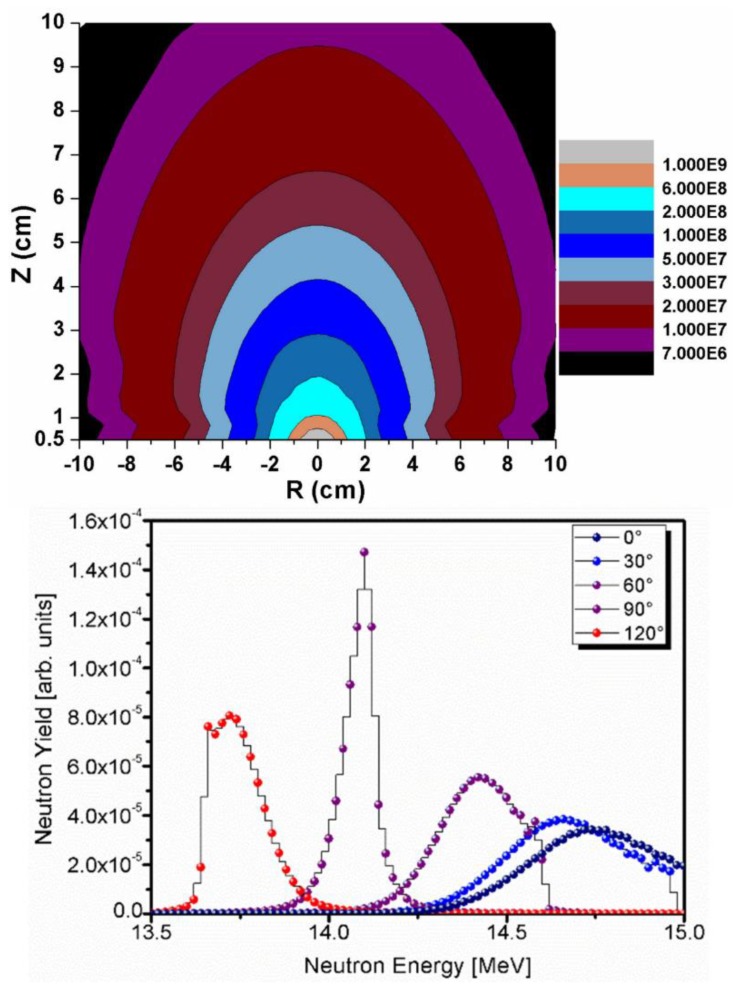
(Upper panel) Simulated Iso-flux loci at FNG relative to a neutron emission rate *Y* = 10^10^ s^−1^; (lower panel) Neutron spectra from FNG source for a selection of five neutron emission angles. Referring to the upper panel, angles are counted anticlockwise.

The neutron emission rate at FNG is known in an absolute way by using the so-called associated alpha particle technique [18]. The fusion reaction in D-T mode produces an alpha particle of 3.5 MeV energy for each 14.1 MeV neutron produced. The number of alpha particles produced during the fusion reactions is measured by means of a silicon detector inside the beam drift tube, and subtending the FNG beam target under a small and accurately defined solid angle. The absolute number of alpha particles measured directly provides the absolute number of neutrons produced by the target. The exact alpha-neutron correlation at the angle subtended by the Silicon Drift Detector (SSD) is accounted for using the well know formula [18] $Y_{a}=\;Y_{n}=\;\frac{4\pi CR_{a}}{\Delta \Omega_{a}}$, $R_{a}$ being an anisotropic correction factor that can be easily calculated, C the experimental alpha particles count rate and $\Delta \Omega_{a}$ the solid angle subtended by the alpha particle detector. Experimental measurements of the neutron flux and energy spectrum, supported by MC simulations carried out with MCNP [19], are within 3% uncertainty. The neutron spectrum obtained at FNG is also well known. This has an angular dependence because the momentum of the incident deuterons impinging onto the target makes the energy of the neutrons varying with the emission angle (Figure 3) [20].

#### 2.1.2. Molybdenum Sample Preparation

**Figure 4:**
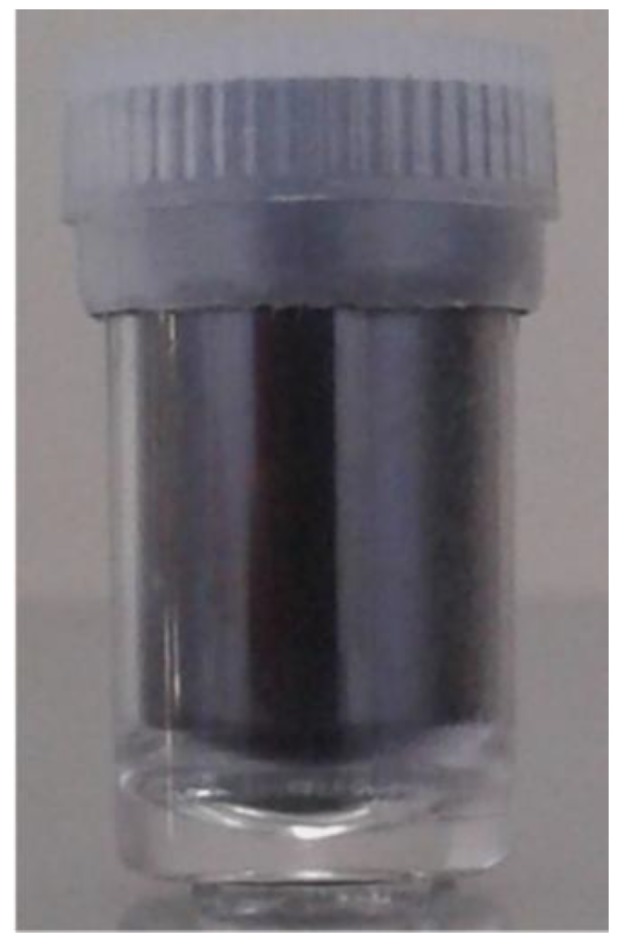
Picture of the natural Molybdenum powder contained into the plexiglass vial used during 14 MeV neutron irradiation at FNG.

The sample used for the 14 MeV neutron irradiation is a metallic powder from Metallwehr Planiee contained into a commercial plexiglass container (Figure 4). To measure the density of the sample when inserted and pressed into the plexiglass holder the following procedure was applied:

1- Three identical plexiglass containers are singularly weighted on a high precision scale (Model RADWAG XA 60/220/X);
2- We put distilled water at a controlled temperature of T = 296 K into the three containers, so to have a well-known value the water density [21];
3- We weighted each container with the water inside;
4- By measuring the water mass inside the container and knowing the water density at that the given temperature, we calculate the inner volume of the container as the average of the three measured values;
5- We put the powder inside one of the three containers, pressing it during filling by means of a small metallic piston;
6- We measure the weight of container and powder and subtracting the average weight of the void container, thus obtaining a molybdenum mass to be irradiated on FNG of 6.5 g;
7- Knowing the powder mass and its volume it is possible to determine the powder density that results to be *d* = (1.840 ± 0.001) g cm^−3^.

#### 2.1.3. Molybdenum Powder Irradiation 

**Figure 5:**
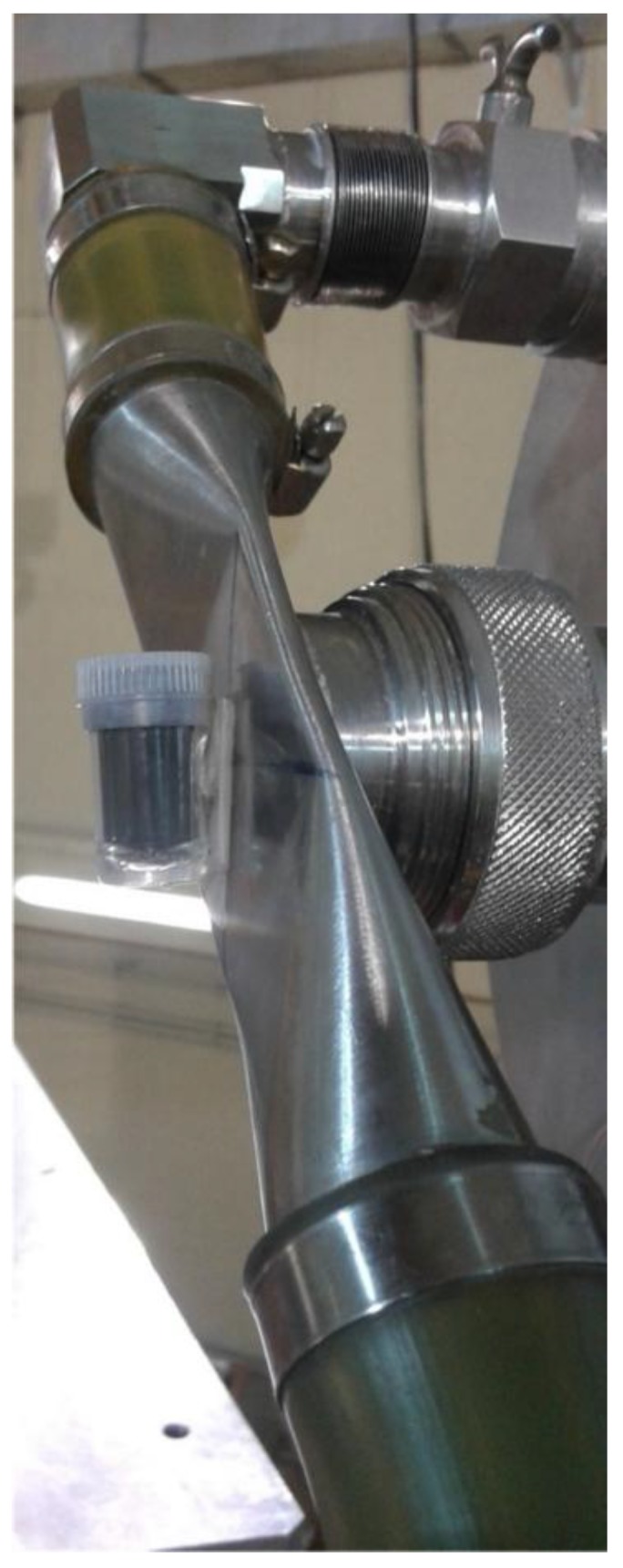
Picture of the experimental setup prepared for the 14 MeV neutron irradiation of the natural Molybdenum powder.

**Figure 6:**
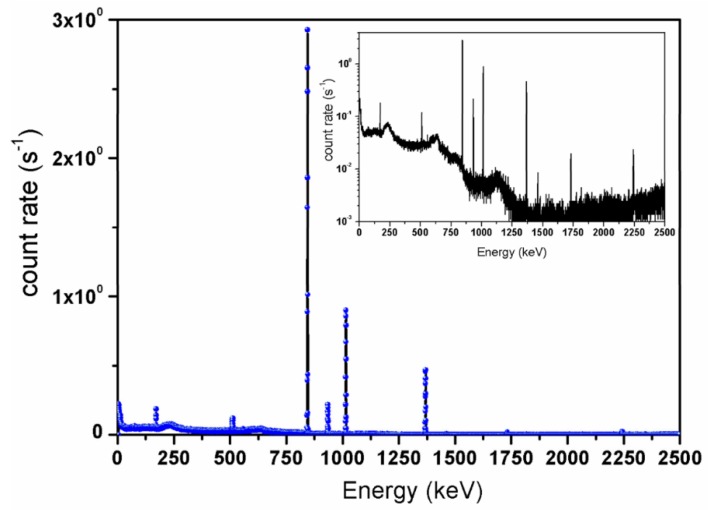
Spectrum from activated Aluminum foil upon 15 min irradiation by 14 MeV neutrons from FNG, recorded with the HPGe available in the FNG laboratory. In the spectrum, the main gamma/ray lines are due to: ^27^Al(n,<)^24^Na reaction channel (*E* = 1368 keV), From the gamma activity of the 1368 keV line 14 MeV neutron flux and source yield are determined. The peaks originating from the ^27^Al(n,p)^27^Mg inelastic reactions are found at *E* = 170,843 and 1014 keV. Also visible in the spectrum is the annihilation peak at *E* = 511 keV.

The sample (i.e., the container with the molybdenum powder) was placed in position close to FNG target (Figure 5). The average flux inside the sample volume was determined by placing onto the plexiglass container an Al activation foil 25 μm thickness and mass of 0.0338 g. Upon irradiation of 14 MeV neutrons, Al undergoes the inelastic reaction ^27^Al(n,α)^24^Na and activates. The activity induced in the foil (Figure 6). Upon neutron irradiation was measured by means of the calibrated HPGe available at the FNG laboratory (Table 1).

This allows a direct measurement of the neutron flux thanks to the relation: $A_{0}=\;N_{T}\int_{\mathrm{Emin}}^{\mathrm{Emax}}\sigma \left(E\right)\cdot \Phi \left(E\right)\cdot dE\cdot \left[1\;-\;\exp\left(-\lambda t_{irr}\right)\right]$ where $N_{T}$ is the number of Al target nuclei in the foil, σ(E) the activation cross section, Φ(E) the neutron spectral fluence rate, λ the daughter nucleus half-life and *t_irr_* the irradiation time.

#### 2.1.4. Gamma-Ray Spectrometry

To quantify at high-metrological level the ^99^Mo produced upon 14 MeV neutron irradiation, the high-energy resolution HPGe detector operating at ENEA-INMRI was used. The detector is coaxial p-type, featuring an efficiency Σ = 0.0045 at 661.6 keV for a point-like source placed at 10 cm from the crystal. The robustness, accuracy and precision of the measurements performed at ENEA-INMRI are guaranteed by their traceability to the primary activity standards [22] developed and maintained in the Institute. The irradiated sample was placed at the reference distance of 10 cm from the detector and the spectrum is recorded for a (live) time of 90,066 s. To estimate the corrections to the efficiency for a volumetric source taking into account the geometry of the sample and its density, an efficiency transfer computation is applied. This was performed by using MC simulation based on GESPECOR4.2 [23] code dedicated to the gamma-ray spectrometry also correcting for coincidence summing. The complete analysis on the recorded spectra is carried out following the procedure described in Ref. [24] to estimate the correct net count rate under the photoelectric peaks associated with the ^99^Mo. All the corrections for background, decay during the measurement time, and decay since the reference date are applied [25].

#### 2.1.5. MC Simulations of the Irradiation of Molybdenum Powder at FNG

**Figure 7:**
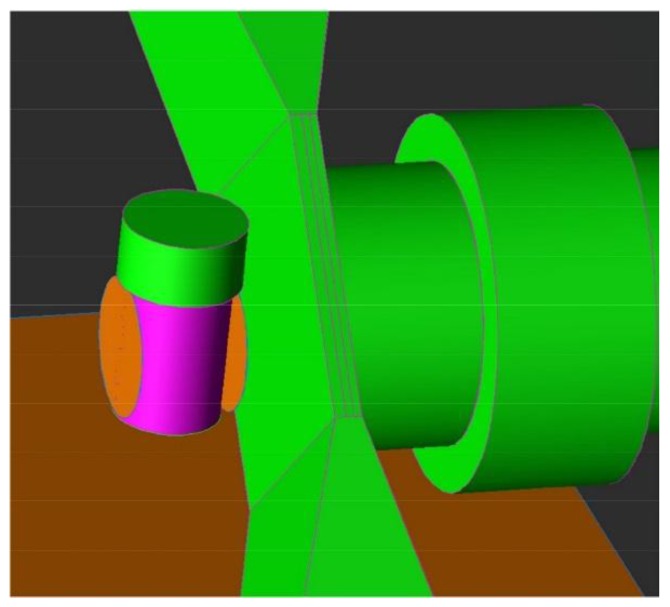
I 3D reproduction of the geometry of the irradiated Moybdenum powder sample as used for the Fluka simulations.

The simulation of the ^99^Mo activity produced after 14 MeV neutron irradiation of natural molybdenum powder is carried out using the Fluka general purpose MC code [13]. The neutron energy fluence spectrum and the geometry of the FNG neutron emitting source are implemented in the Fluka simulation by a proper user routine, written in Fortran, which is compiled and linked to the executable. The nuclear data for molybdenum used by Fluka are the EFF–2.4 (The European Fusion File) [13].The geometry and the materials of the irradiated sample are fully implemented into the model (Figure 7): a Plexiglascylindrical container filled with natural molybdenum powder, made of a mixture of isotopes according the natural composition (Table 2), but with assigned density corresponding to the measured one (1.940 ± 0.001) g cm^−3^. The FNG bunker is not considered in the simulation since irrelevant to the assessment of the result: in fact, the scattered neutron fluence rate at the irradiation point is estimated to be almost four orders of magnitude lower than that at 14 MeV. The radionuclides produced and their activity are calculated with the RESNUCLEI card that, together with the “IRRPROFI” and the “RADDECAY” cards, allows to activate the generation and transport of the decay products, according a given primary particle emission rate and a specific irradiation time. The “IRRPROFI” card also enables response to complex duty cycles, i.e., a sequence of multiple beam-on and beam-off periods also with different intensity. The actual irradiation beam profile of the experimental test carried at FNG is reproduced: an irradiation time of 900 s with a constant neutron emission rate of 2.89 × 10^10^ s^−1^. The average neutron fluence rate inside the sample is evaluated to be 1.1 × 10^9^ cm^−2^ s^−1^, while the neutron current across the sample surface is estimated 3.8 × 10^8^ cm^−2^ s^−1^, in agreement within a factor 1.3 with the experimental value provided by the activation measurement of the Aluminum foil, on the external surface of the Mo sample container (also introduced in the simulated setup). By means of Fluka, it is possible to directly calculate the integral activity or the activity per unit volume of each radionuclide produced during the irradiation time as well as at different times after the irradiation (the so-called “cooling times” by using the “DCYSCORE” card) and to follow and transport the decay products. Fluka estimates that the ^99^Mo specific activity produced at FNG at the end of the irradiation is 2.50 kBq g^−1^ with 1% statistical error. The overall combined statistical uncertainty is 11% of the predicted value resulting from the uncertainties of: (1) the nuclear data for the cross section at 14 MeV used in Fluka; (2) the FNG neutron emission rate; (3) the sample positioning with respect to the neutron source and (4) MC simulation statistics. Thus, the 2.32 kBq g^−1^, experimentally measured by means of gamma-ray spectrometry, well compares to the calculated one (2.50 kBq g^−1^) within the error. The good agreement between the Fluka results and the experimental measurements of the FNG test for all the produced radionuclides whose activities is measured at the INMRI (Table 3), benchmarks our model showing the ability of describing in a realistic way both the 14 MeV neutron fusion source and the molybdenum powder, relying on the physical models and nuclear data implemented in the Fluka code. This makes us confident in being able to perform reliable predictions of the ^99^Mo produced by 14 MeV neutron source on enriched ^100^Mo powder target.

### 2.2. Extraction of the Pertechnetate and Quality Controls 

**Figure 8:**
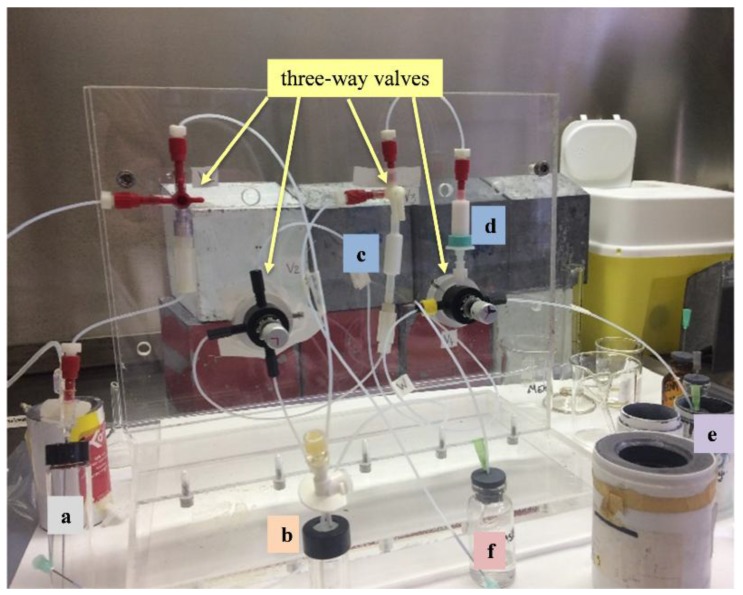
Picture of the automatable solvent extraction module. (**a**), In this vial occurs the dissolution with H_2_O_2_ in H_2_O of irradiated natural molybdenum powder and the basification with NaOH. (**b**), Vial for solvent extraction of pertechnetate with MEK from the aqueous alkaline solution. (**c**,**d**), Silica and alumina cartridges respectively, used for the purification of the extracted pertechnetate. (**e**), Vial (placed inside a shielded lead container) used for the elution of sodium pertechnetate [^99m^Tc]NaTcO_4_ from the alumina column with saline. (**f**), Vial used for waste.

The automatable module developed and properly optimized for this project (Figure 8) involves five main steps: (1) dissolution of the irradiated target and basification of the solution; (2) solvent extraction of pertechnetate with MEK from the aqueous alkaline solution; (3) separation of the MEK phase, containing pertechnetate, from aqueous solution containing molybdate and by-products; (4) purification of the extracted pertechnetate by column chromatography with a silica and an alumina cartridges and finally; (5) elution of sodium pertechnetate [^99m^Tc]NaTcO_4_ from the alumina column with saline [26,27,28]. The dissolution, extraction and purification processes were performed the day after sample irradiation to achieve the equilibrium between ^99m^Tc and the parent nuclide ^99^Mo. The procedure was carried out in a shielded radiochemistry hood by means of the automatable module. The procedure takes about 45 min, comprehensive of the dissolution step, and allows to obtain ^99m^Tc in high yield with minimum activity losses. This technique was also recently applied for the high yield separation of ^99m^Tc directly produced by proton irradiation with cyclotron on an enriched ^100^Mo sample [26,27]. A detailed quality control analysis of the final pertechnetate solution was performed, to determine isotopes and impurity amounts, Mo breakthrough, percentage of organic solvent etc. The quality of the product is evaluated as radiochemical purity by radio TLC, radionuclidic purity by gamma-ray spectrometry with HPGe detector and chemical purity by colorimetric strip tests, gas-chromatography and inductively coupled plasma mass spectrometry (ICP-MS). The chemical and radiochemical purity values are also assessed (Table 3 and Table 4).

Chemical analysis of a saline solution aliquot, containing ^99m^Tc from extraction and separation procedure, is performed by means of an ICP-MS equipped with Octapole Reaction System (ORS) fueled with He collision cell and H_2_ cell gas, to reduce spectrometric interferences both polyatomic and isobaric. ICP-MS analyses is carried out using an Agilent ICP-MS (Inductively Coupled Plasma Mass Spectrometry), Agilent 7700× equipped with: a collision/reaction Cell ORS3 (Octapole Reaction System, 3rd generation cell design, Agilent, Santa Clara, CA, USA), a concentric quartz MicroMist Nebulizer as a sample introduction system. ICP-MS is optimized with a multiple standard tuning solution 10 μg/L produced by Agilent Technologies Spa (Table 5). The aliquot described previously is evaporated to dryness. The residual dissolved in 1 mL of nitric acid conc. and then added with ultrapure water (18 MΩ) to 10 mL total. This solution is analyzed directly with ICP-MS first by a scanning method (SCAN) on all the masses (from M/Z = 3 to 254, M ad Z being the atomic mass and number, respectively) and then on each mass corresponding to the significant values obtained from the previous scan. The quantitative analysis is made after subtraction of the Blank (background + water + acid) and comparing the Counts with Reference Standard Solutions of known concentration (Table 4). The concentrations are extremely low, and they can be referred as impurities contained in the solvents used (MEK, Water, Nitric Acid) and/or laboratory materials used during the extraction and preparation (vials, plastics, tubings, …).

### 2.3. Predictions of the ^99^Mo Activity Producible at the NSFS and Thermo-Physical Analysis of the ^100^Mo Targets

**Figure 9:**
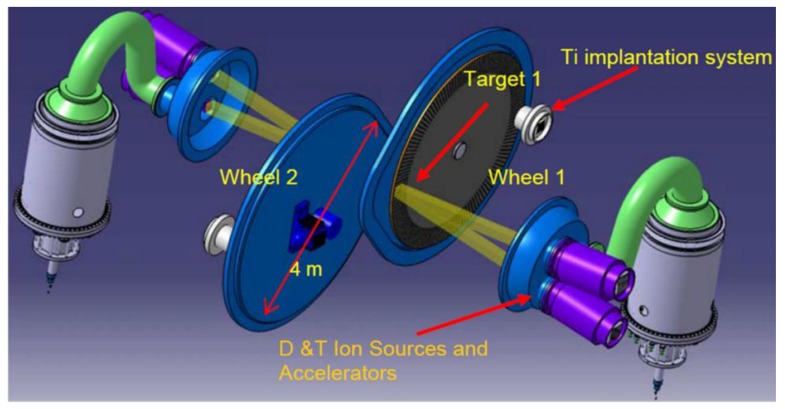
Schematic view of the double target NSFS. Two deuteron beams are directed onto a tritium-loaded target where fusion reactions take place. The tritium beams continuously implant tritium onto the target to maintain reaction rate.

Several MC simulations have been performed, to predict the ^99^Mo total activity obtainable with targets of different geometry, made of pure ^100^Mo, for several irradiation profiles (i.e., neutron rates and time windows of irradiation), at different cooling times after the neutron beam shutdown. The activities of all the other radionuclides produced have been also estimated at the end of the irradiation. To provide “reliable” and “conservative” estimations of the ^99^Mo production capabilities at NSFS, the most important physical operative constraints that could actually limit the operation of NSFS are identified. The irradiation modalities are, in fact, mainly related to the thermo-mechanical issues that a ^100^Mo target can experience when the maximum NSFS neutron emission rate is used for a long irradiation time (without using any active cooling system), attempting to maximize the ^99^Mo activity that could be produced. The available irradiation room considered is in between the two NSFS rotating targets also referred to as the NSFS wheels (Figure 9). 

**Figure 10:**
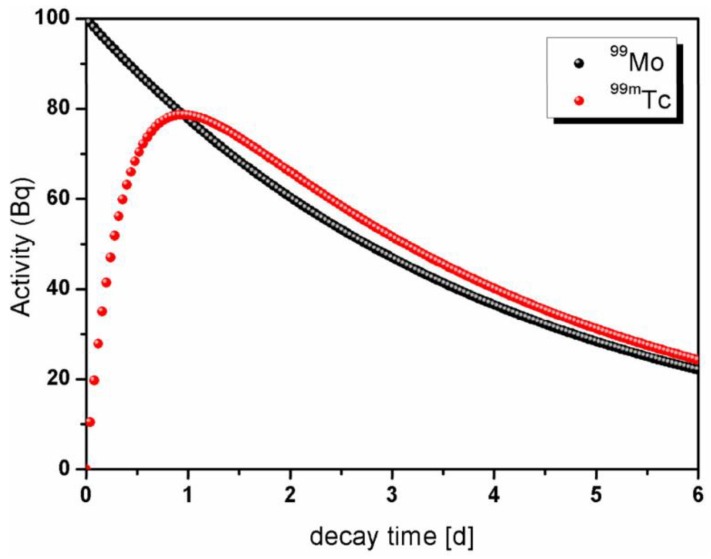
Plot of the transient equilibrium between ^99^Mo and ^99m^Tc. From the trend it is clear that the optimal irradiation time is of about 22 h.

An operative neutron emission rate of 4 × 10^15^ s^−1^ and an optimized irradiation time of 22 h are assumed in our simulations, the latter being identified on the base of the decay time curve of the transient equilibrium between ^99^Mo, precursor, and ^99m^Tc, daughter (Figure 10). 

**Figure 11:**
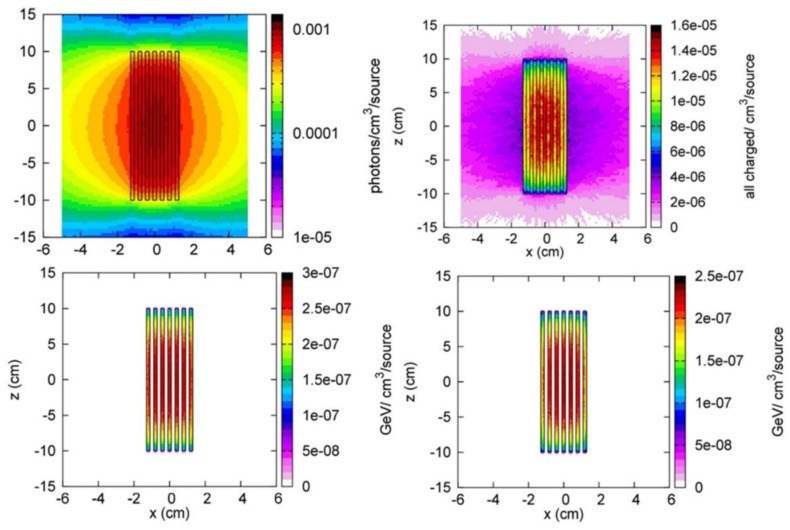
^100^Mo segmented target used to provide a realistic prediction of ^99^Mo production at NSFS (2 × 10 × 20 cm^3^): (upper left): gamma density; (upper right): total charged-particle density; (lower left): total energy deposition; (lower left) electromagnetic energy deposition. Data obtained by using MC Fluka code. All the results are per unit primary particle. The estimations have been obtained by Fluka (version 2011.2c.3) code, assuming a uniform and isotropic irradiation of 14 MeV neutrons coming from the NSFS wheel surfaces.

The energy deposited in the target, which causes the heating of the target itself, is mainly due (more than 80%) to the electromagnetic radiation ensuing the inelastic interaction of neutrons with the target nuclei (Figure 11). The temperature distribution inside the target is evaluated taking into account the power density deposition as derived from the Fluka results and the heat exchange by irradiation. The material of the coating for the two NSFS wheels is assumed to be Titanium at the uniform temperature of 693 K and both polished and oxidized cases for the surface status are considered. A uniform temperature equal to the maximum value expected on the NSFS wheels at the nominal power (693 K) is also a conservative assumption since it does apply the highest temperature to all the surfaces interfaced with the ^100^Mo target, disregarding the thermal gradient that could make the heat exchange more efficient, further lowering the maximum temperature. The ANSYS thermal calculations enable the accomplishment of a transient analysis of the temperature field inside the NSFS irradiation chamber during all the irradiation time, so to get results as accurate as possible, all the thermo-physical properties of the ^100^Mo (heat capacity, thermal conductivity, emissivity) have been introduced as a function of the temperature. In all the estimations the transversal area of the ^100^Mo targets (either the bulk or segmented ones) is assumed to be 10 × 20 cm^2^. Both bulk and segmented targets are analyzed: the segmented targets are supposed to be obtained by arranging, side by side, 200 tiles of 1 cm × 1 cm transversal area on a rectangular frame 20 cm wide and 10 cm height. The heat power density inside the ^100^Mo targets is activated by the “BFE” (Body Force Element) command to define body loads, applied according the spatial profile of the energy deposition, on all the elements in which the ^100^Mo target geometry has been discretized. The discretization of the ^100^Mo volumes is done using SOLID70 elements since this kind of element is suitable for three-dimensional steady-state as well for transient thermal analysis. The surface to surface radiation is set assigning the proper emissivity by means of the surface load command “SF, RDSF” and specifying the enclosure that defines the surfaces involved in the radiation heat exchange (i.e., in case of a bulk ^100^Mo target only two enclosures are defined, while for a segmented target with n plates, the number of the enclosure considered is n + 1, neglecting the radiation through the lateral thickness since its emitting surface is, depending on the targets, from 1 to 2 orders of magnitude smaller than the target transversal ones). Several ^100^Mo target configurations have been studied and for all these the ^99^Mo production at the end of irradiation and the temperature field have been computed, seeking the optimal target configuration that can maximize the ^99^Mo production keeping the internal temperature well below the fusion considered as reasonable required operative constraints. At the end of our analysis, the 7 plates segmented target (2 mm thick, 10 × 20 cm^2^ area and 390 g mass) is assumed as a reference “realistic” target (see Table 6), since able to provide an optimized and representative ^99^Mo production rate in NSFS at its full nominal power, fulfilling all the thermal constraints. This target has been identified as result of technical considerations that consider: (a) the geometry and size of the irradiation chamber of NSFS; (b) the need of using thin plates despite bulk massive targets to facilitate the ^99^Mo extraction process and (c) the requirement to work with as low as possible temperature in the irradiated materials.

Nevertheless, it is not excluded that more suitable shapes and thicknesses could be found and used, since the optimization process of the target will be completely defined only after the final assessment of the design and operative parameters of the NSFS facility.

## 3. Results and Discussion

### 3.1. ^99^Mo Production by Means of 14 MeV Neutrons 

FNG is an accelerator-driven continuous neutron source which relies on the fusion reaction:

 D + T → α (3.5 MeV) + n (14.1 MeV) 

FNG produces almost monochromatic neutrons with a nominal maximum neutron emission rate of 1011 s^−1^, well determined by means of an absolute measurement based on the so-called associated alpha particle technique [29]. The natural molybdenum powder sample is irradiated at FNG for 15 min with a measured neutron emission rate of 2.89 × 10^10^ s^−1^. In these experimental conditions, a ^99^Mo specific activity of (2.32 ± 0.05) kBq g^−1^ is achieved at the reference time [8,25].

This result is used to benchmark the prediction of the activity obtained by MC calculations performed by means of the Fluka code. The relevant role of MC simulations relies on the possibility of estimating the specific activity taking correctly into account the geometrical and physical parameters that cannot easily be described by analytical methods. In fact, we can evaluate the neutron spatial distribution and all the involved reactions (transport and nuclear interactions) within extended irradiation targets, including their actual chemical and isotopic composition. It is worth stress that a competing ^99^Mo production mechanism in the sample used for the tests and simulations that is ^98^Mo(n,Υ)^99^Mo provide a negligible contribution or two reasons: (1) the thermal neutron component at the irradiation position is about three order of magnitude lower in intensity than the 14 MeV component; (2) the ^98^Mo(n,Υ)^99^Mo reaction induced by 14 MeV neutrons feature a cross section that is found to be three order of magnitudes lower than the ^100^Mo(n,2n)^99^Mo one. The result for the ^99^Mo specific activity obtained by means of the Fluka calculation is (2.50 ± 0.27) kBq g^−1^ that well compares to the experimental value. The relative standard uncertainty on the calculated value is obtained by combining all the uncertainties due, mainly, to: simulation statistics, ^100^Mo(n,2n)^99^Mo cross section data and FNG neutron yield. Moreover, Fluka allows the building up of a dynamic picture of all the occurring decay processes of the whole set of radionuclides produced within the irradiated target. The specific activity of each calculated main impurity is shown in Table 3, where the values are compared to those measured by gamma-ray spectrometry.

All the measured activities are traceable to the National Activity Standards maintained at the Italian National Institute of Ionizing Radiation Metrology (ENEA-INMRI) [25], thus minimizing the uncertainty to the experimental data.

The set of measurements carried out at ENEA are a profitable step to build and assess a valuable procedure for the computational and experimental verification of the ^99^Mo production using 14 MeV neutrons, in controlled and reliable way. The peculiarities of the applied procedure can be summarized as follows:

- use of a D-T fusion neutron source well-characterized in energy and emission rate;
- optimization of the experimental setup and irradiation time by MC calculations;
- exploitation of metrological tools to achieve high levels of accuracy and traceability for the ^99^Mo and ^99m^Tc activity measurements and MC benchmarking purposes.

### 3.2. Separation and Purification Process

A further step in our experiment is the extraction of the pertechnetate, i.e., the potentially injectable ^99m^Tc radiopharmaceutical for SPECT. This is a mandatory step to completely assess the viability of the whole process from ^99^Mo production toward a usable radiopharmaceutical.

**Figure 12:**
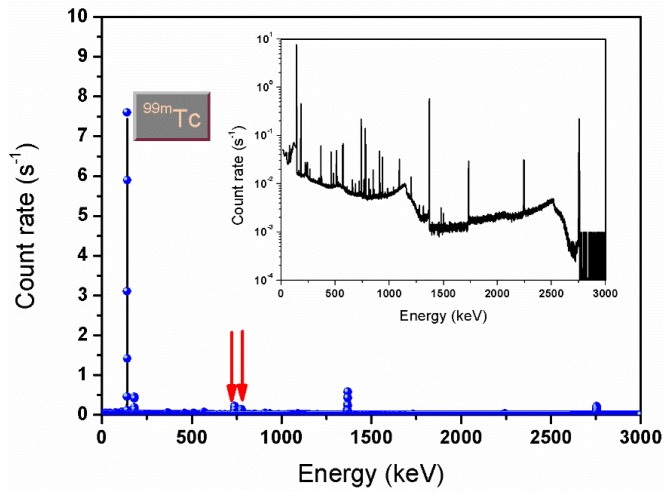
Gamma-ray spectrum recorded by the INMRI-ENEA HPGe detector from the irradiated Mo powder at FNG facility. Red arrows indicate the gamma-rays from the ^99^Mo decay.

**Figure 13:**
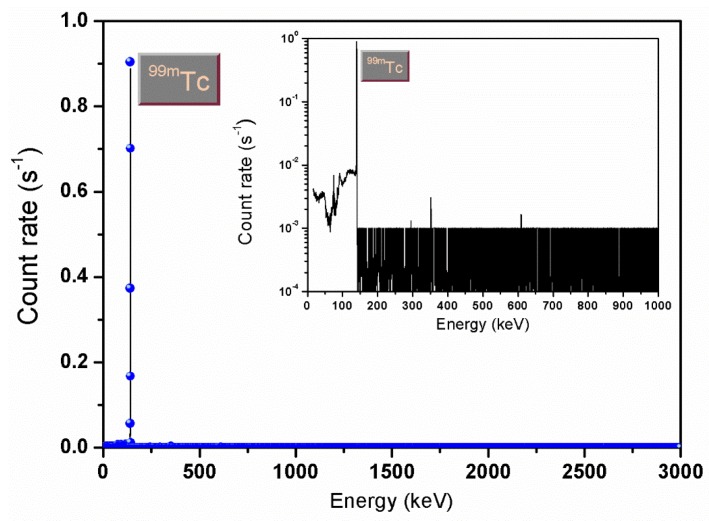
Gamma-ray spectrum recorded by the INMRI-ENEA HPGe detector from the extracted pertechnetate.

To separate and purify the ^99m^Tc obtained as decay product of the ^99^Mo produced at FNG, an automatable extraction module based on solvent extraction has been developed. The choice of this extraction procedure relies on the high efficiency demonstrated on this kind of application by Chattopadhyay et al. [30,31] and Martini et al. [32,33]. Indeed, the selected affinity of pertechnetate for the organic solvent methylethylketon (MEK) allows a high pertechnetate yield extraction (greater than 90% [20]) from an aqueous alkaline phase in which molybdate and other by-products remain in solution. Furthermore, the combination of solvent extraction with column chromatography purification helps in decreasing chemical and radiochemical contamination. Finally, the module enables the multiple extraction procedure by saving the aqueous alkaline solution containing ^99^Mo that continues decaying to ^99m^Tc. The use of this extraction/purification technique enables the obtaining of a final product in compliance with the threshold values imposed by the Pharmacopoeia for product injectability [34]. Figure 12 and Figure 13 show, respectively, the gamma-ray spectra recorded by the High-Purity Germanium (HPGe) detector after the 14 MeV neutron irradiation of the molybdenum powder sample at FNG and after the extraction of the pertechnetate. The inserts show the same spectra but in logarithmic scale to appreciate the intensities.

The two spectra show how the whole separation and purification procedure provides a pertechnetate free of any impurities.

### 3.3. Perspectives for NSFS

The benchmarked MC model for the 14 MeV neutron irradiation of the powder sample at FNG enables the making of confident predictions of the nominal ^99^Mo activity obtainable at NSFS. The latter is an intense 14 MeV neutron source based on D-T fusion reactions thoroughly described in Refs^.^ [14,15,16]. The nominal NSFS neutron emission rate, in continuous state, is about 4–5 × 10^15^ s^−1^, i.e., more than 104 times higher than the nominal value available at FNG.

Taking into account the main thermal constraints on the maximum allowed temperature into the molybdenum irradiation target (i.e., keeping the target below the melting point) and the current available manufacturing options on the ^100^Mo enriched sintered disks (plates of the order of a few millimeters thickness), a series of target configurations in the NSFS irradiation vacuum chamber have been studied and evaluated (from bulk configurations to different solutions of segmented targets). The aim is to provide a realistic and representative estimation of the predictable weekly ^99^Mo activity production, considering the NSFS nominal neutron emission rate.

A conservative, and rather pessimistic, irradiation duty cycle (DC) of about 50% (i.e., 22 h dedicated to irradiation and 24 h for setup activities). An irradiation time of 22 h has been assumed. on the base of the decay time curve of the transient equilibrium between ^99^Mo, precursor, and ^99m^Tc, daughter.

The thermal calculations have been performed by the ANSYS finite element code [35]. The heat power deposition inside the target has been introduced according to the Fluka simulation results on the total energy density deposition. The heat exchange by irradiation between the target (also considering all its surfaces when segmented) and the NSFS neutron emitting surfaces has been also simulated. A realistic reference target is considered that is made of seven equally spaced plates of 2 mm thickness and 10 × 20 cm^2^ transversal cross section, for a total enriched ^100^Mo mass of about 2.7 kg. The ANSYS calculations show that this target can be safely irradiated continuously for 22 h, since the maximum temperature is predicted to be 732 K, well below the molybdenum melting point (T = 2833 K). The ^99^Mo produced at the end of the irradiation in this case is expected to be 66 TBq, that is almost 200 TBq in a week, according to the assumed conservative DC.

In a more optimistic (and realistic) DC scenario, with only a few hours interval between one target irradiation and the following one, the total weekly amount of ^99^Mo achievable should be almost doubled. In terms of 6-day Ci activity EOP (see Table 7), the conservative estimations in the case of the segmented target, correspond to 44 TBq and 88 TBq per week for DC = 50% and 100%, respectively.

## 4. Conclusions

From the values of the nominal weekly activity, *NA***_w_**, calculated in the aforementioned scenarios, we make a comparison between the nominal 6-day Ci-EOP activities achievable in a selection of representative research nuclear fission reactors and NSFS, as reported in Table 8 [36].

**Figure 14:**
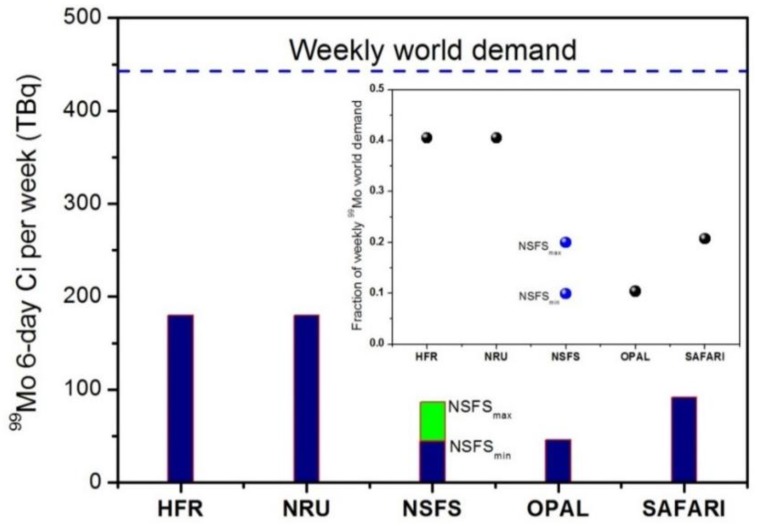
(Color online) ^99^Mo 6-day Ci production for the different facilities listed in Table 8.

In Figure 14, the values listed in Table 8 are reported on a plot for an easier visual inspection, where the weekly nominal world demand (444 TBq) is indicated as the reference quantity. 

NSFS_min_ and NSFS_max_ indicate the lower (44 TBq) and upper limit (88 TBq) of ^99^Mo achievable production, respectively. The broken line marks the 6-day Ci weekly world demand. The inset shows the fraction of the nominal ^99^Mo weekly world demand covered by the different irradiation sites.

It is found that the range of ^99^Mo production in NSFS is obvious if compared to that of the main (but aging) nuclear fission reactors that cover quite a large fraction of the present ^99^Mo market.

It is also worth remarking that although the ^99^Mo production mechanism with D-T fusion reactions is different with respect to the current one relying on the nuclear fission reactors, the overall distribution chain from the production site to the final users is expected to remain unchanged. Moreover, the ^100^Mo that did not interact during the first irradiation can be recovered and further utilized for new irradiation rounds.

In summary, we investigated the ^100^Mo(n,2n)^99^Mo reaction in view of the possible production of ^99m^Tc radiopharmaceutical, as one of the possible routes that are alternative to the ^99^Mo production via ^235^U fission. To this aim, we performed a natural molybdenum powder target irradiation using 14 MeV neutrons from the accelerator-driven FNG facility, under a neutron emission rate of about 2.9 10^10^ s^−1^ for 15 min. 

We obtain a specific ^99^Mo activity of (2.32 ± 0.05) kBq g^−1^, a measurement traceable to the National Activity Standards of ENEA-INMRI. Furthermore, MC simulations based on the Fluka code and benchmarked by the experimental data collected at FNG enable a quantitative and reliable prediction of the ^99^Mo weekly activity that may be provided by NSFS, designed to deliver a neutron emission rate of about 10^15^ s^−1^. It can be stated that NSFS can possibly produce a large weekly activity of ^99^Mo, fulfilling the requirement expressed by international organizations (IAEA, OECD) of finding a viable, suitable and safe production chain alternative to that based on ^235^U fission operative at nuclear fission reactors.

This work highlights that a robust alternative can be achieved using intense 14 MeV neutron fields such as the one expected to be achieved at NSFS. In the perspective, a high intensity 14 MeV neutron source such as New Sorgentina Fusion Source (NSFS), may secure the long-term supply of ^99^Mo at global level and thus the production of high-purity ^99m^Tc for medical applications.

## Figures and Tables

**Table 1:** Efficiency values of the HPGe used at the FNG laboratory. These values allow to determine from the measurement of the activity of the Al foil the neutron flux at the foil position that is the neutron flux used to irradiate the Molybdenum powder. The uncertainty on the efficiencies is 3%.

Energy (keV)	Efficiency
59.54	6.3948 × 10^−3^
80.99	1.1430 × 10^−2^
121.78	1.2462 × 10^−2^
244.67	9.4432 × 10^−3^
276.40	9.2965 × 10^−3^
302.85	8.7134 × 10^−3^
344.30	7.6811 × 10^−3^
356.01	7.8276 × 10^−3^
383.84	7.4923 × 10^−3^
411.80	6.9161 × 10^−3^
661.60	5.0448 × 10^−3^
778.90	4.3317 × 10^−3^
867.39	4.0252 × 10^−3^
964.00	3.8244 × 10^−3^
1085.80	3.5155 × 10^−3^
1112.07	3.5334 × 10^−3^
1173.25	3.3827 × 10^−3^
1332.50	3.0695 × 10^−3^
1408.08	2.9852 × 10^−3^

**Table 2:** Isotopic composition of the natural molybdenum powder.

Radionuclide	Abund (%)
^92^Mo	14.8
^94^Mo	9.3
^95^Mo	15.9
^96^Mo	16.7
^97^Mo	9.6
^98^Mo	24.1
^100^Mo	9.6

**Table 3:** MC predicted and measured specific activity for other relevant radionuclides than ^99^Mo generated after 14 MeV neutron irradiation of the natural Molybdenum powder.

Z	Radionuclide	SA_MC_ (*) (Bq g^−1^)	SA_Exp_ (Bq g^−1^)
40	^89^Zr	46.35 ± 1.6%	48 ± 4.9%
	^95^Zr	0.96 ± 5.5%	0.97 ± 6.4%
	^97^Zr	16.1 ± 9.1%	16.6 ± 5.8%
41	^93^Nb^m^	39.1 ± 1.8%	40 ± 4.5%
	^95^Nb	189.7 ± 2.5%	187 ± 1.8%

(*) Calculated values with simulation statistical uncertainties.

**Table 4:** Radiochemical (RCP) and chemical (CP) purity values of the [^99m^Tc]TcO_4_^−^ solution obtained at the end of the separation and purification procedure.

Quality Controls	RCP	CP			
	[^99m^Tc]TcO_4_^−^	pH	Mo	Al	MEK
Experimental	>99%	5	<5 ppm	<5 ppm	<0.0005% (*v*/*v*)
EU Pharmacopoeia	≥95%	4–8		<5 ppm	<0.5% (*v*/*v*)

**Table 5:** Impurities concentrations in the pertechnetate solution after extraction and purification. Concentration is given in in ppb (part of billion).

Element	M (amu)	C (μg/L)
Be	9	2.84
In	115	0.18
Sm	147	0.08
Tb	159	0.05
Er	166	0.07
Yb	172	0.03
Re	185	3.72
Pb	208	0.29

**Table 6:** Optimized operation condition and data acquisition parameters for the ICP mass spectroscometry.

Forward Power	1550 W
Nebulizer gas	1.07 L/min
Carrier gas	1.09 L/min
Sample flow	100 µL/min
Torch	single piece, quartz
Nebulizer	Concentric quartz MicroMist Nebulizer
Spray chamber	quartz impact bead
Makeup gas	0.00 L/min
Option gas	0.0%
Collision Cell gas (He)	5.0 mL/min and 10.0 mL/min (in High-Energy Mode)
Reaction Cell gas (H_2_)	6.0 mL/min
Octapole bias	−18.0 V
KED	3.0 V
Dwell time	0.3 s

**Table 7:** 6-day Ci (EOP) ^99^Mo activity scenarios, achievable with a representative selection of ^100^Mo target configuration, for an irradiation time of 22 h and a Duty Cycle of 50%; BT refers to bulk target, ST refers to the segmented case. *M*_Mo_ is the ^100^Mo mass, θ the ratio between the maximum sample temperature to the Molybdenum melting temperature and *NA*_w_ is the nominal weekly activity at the End Of Process (EOP).

Scenario	Δ [cm]	*M*_Mo_ [Kg]	θ	*NA*_w_ EOP [TBq]
BT *	1.5	2.8	0.30	34
ST *	0.2 ** (7 Plates) 0.1 ** (15 Plates)	2.7 2.8	0.25 0.19	44 37

* thickness of the single segment (2 mm pitch between the centers of two consecutives plates); ** All samples have the same transverse area of 10 × 20 cm^2^.

**Table 8:** Nominal weekly activities (6-day Ci-EOP) for selection fission reactors and the accelerator-driven 14 MeV neutron source NSFS.

Fission Facilities	*NA*_w_ [Bq]
HFR-PETTEN [37]	1.8 × 10^14^
SAFARI-PELINDABA [38]	9.2 × 10^13^
NRU-CHALK RIVER [39]	1.8 × 10^14^
OPAL-SYDNEY [40]	4.6 × 10^13^
D-T Fusion source	
NSFS [14,15,16]	(4.4 ÷ 8.8) × 10^13^
